# New Generation Automatic Massage Chairs for Enhancing Daytime Naps: A Crossover Placebo-Controlled Trial

**DOI:** 10.3390/healthcare13182291

**Published:** 2025-09-12

**Authors:** Ilias Ntoumas, Nikolas Antoniou, Christoforos D. Giannaki, Fotini Papanikolaou, Aggelos Pappas, Efthimios Dardiotis, Christina Karatzaferi, Giorgos K. Sakkas

**Affiliations:** 1 Department of Physical Education and Sport Science, School of PE, Sports and Dietetics, University of Thessaly, 42100 Trikala, Greece; intoumas@uth.gr (I.N.); adoniounikolas@gmail.com (N.A.); papanikolf@gmail.com (F.P.); apappa66@yahoo.com (A.P.); ck@uth.gr (C.K.); 2 Department of Life Sciences, School of Life and Health Sciences, University of Nicosia, Nicosia 2417, Cyprus; giannaki.c@unic.ac.cy; 3 Research Centre for Exercise and Nutrition (RECEN), Nicosia 2417, Cyprus; 4 Department of Neurology, School of Medicine, University of Thessaly, 41110 Larisa, Greece; edar@med.uth.gr

**Keywords:** daytime nap, massage chair, EEG, sleepiness scale, handgrip, heart rate

## Abstract

**Background/Objectives**: Modern technology is transforming the field of massage, enhancing relaxation and wellness through innovative devices. The aim of the present study was to examine the effect of various massage protocols available using an automatic electric massage chair (AEMC) prior to daytime napping on relaxation and indices of sleep quality. **Methods**: This study is a randomized, single-blind, placebo-controlled, four arm, interventional clinical trial. A total of 12 healthy individuals (21.8 ± 2.2 years, 6 F/6 M) were randomly assigned to four different groups: (1) the control (CON) session involving a 30 min rest on an automatic switch-off massage chair, (2) the easy-sleep (ES) massage session designed to promote sleep, (3) the fatigue-recovery (FR) massage session designed to reduce muscle fatigue, and (4) the worker-mode (WM) massage session designed to promote muscle relaxation. During the four sessions, participants sat in the massage chair for 30 min, followed immediately by an additional 30 min period of lying down on a standard double bed. Brain activity was monitored using a polysomnography EEG system, while validated tests and questionnaires assessed vitals and the state of relaxation. **Results**: The ES massage significantly reduced muscle tone by 12% and heart rate by 22% (*p* = 0.008 and *p* = 0.007, respectively). Additionally, it increased subjective sleepiness by 4.5% and sleep efficiency by 5.7% compared to the results for the control condition (*p* ≤ 0.005). **Conclusions**: It is evident that the use of an AEMC can reduce tension and improve feelings of relaxation. The easy-sleep program seems to be a promising non-pharmacological approach for enhancing relaxation and promoting daytime sleep, acting as a non-pharmacological tool to reduce stress, improve sleep quality, and promote workplace well-being. The trial was registered as NCT06784700.

## 1. Introduction

The fast-paced nature of the modern lifestyle often contributes to increased stress before bedtime that negatively impacts many aspects of sleep quality, highlighting the need for interventions that promote relaxation. One of those interventions is daytime napping. Naps are typically defined as short periods of sleep, usually lasting from 10 to 30 min, taken during the day to supplement nighttime rest. Naps can enhance cognitive and physical performance, improve mood, and increase alertness, making them an effective strategy for mitigating all-cause fatigue [1,2]. Bibliographic evidence suggests that brief afternoon naps can provide numerous benefits for health and cognitive function, enhancing alertness, mood, and performance on demanding tasks. While the effects of sleep interventions have been widely investigated, the role of the various relaxation objects that promote sleep or calmness remain less thoroughly explored. Recent studies have indicated that ergonomic chairs, bean bags, or specialized sleep chairs may promote physiological relaxation through muscle unloading and activation of the parasympathetic nervous system, leading to improved sleep quality and longer sleep duration [3]. These findings highlight the significance of the sleep environment in facilitating recovery and underscore the potential beneficial effect of using specialized seating as a supplementary tool for managing fatigue and stress in everyday life, especially in the workplace.

Another potential method to mitigate pre-sleep stress is massage therapy [4]. Massage can reduce stress and anxiety [5,6,7], contributing to better sleep quality [8]; however, this approach may not be feasible for everyday usage due to differences in sleep schedules and financial constraints. A more convenient and accessible alternative could be the usage of automatic electric massage chairs (AEMCs).

AEMCs offer a non-pharmacological alternative for achieving relaxation and improving sleep quality, particularly for individuals with high working stress levels [9]. Many studies from the last decade demonstrate that massage chairs provide significant physical and mental health benefits, including stress reduction, alleviation of mental fatigue and pain, enhanced muscle flexibility, and overall improvement in quality of life [10].

Massage chairs offer many automatic massage options for alleviating muscle tension and reduce fatigue, contributing to improved functionality and well-being, as well as promoting sleep [11]; however, scientific evidence to support these claims are very scarce, and most claims are based on manufactures’ non-published data. Indeed, the effects of massage can vary significantly depending on factors such as the targeted body region, the type of massage technique, the applied pressure, and the setting (private vs. public place). Thus, while some general benefits have been reported, specific outcomes may differ between massage chair protocols. Furthermore, scientific evidence supporting these claims remains limited, as many reported benefits are derived from unpublished manufacturer data.

Therefore, the purpose of the present study was to examine the effectiveness of various automatic massage protocols available οn AEMCs in regards to relaxation and indices of sleep quality in healthy individuals prior to daytime nap.

Based on the design and aims of the current study, it was hypothesized that the ES-massage would be the most effective condition in promoting relaxation and improving sleep quality among the four protocols tested.

## 2. Materials and Methods

### 2.1. Subjects

**Figure 1:**
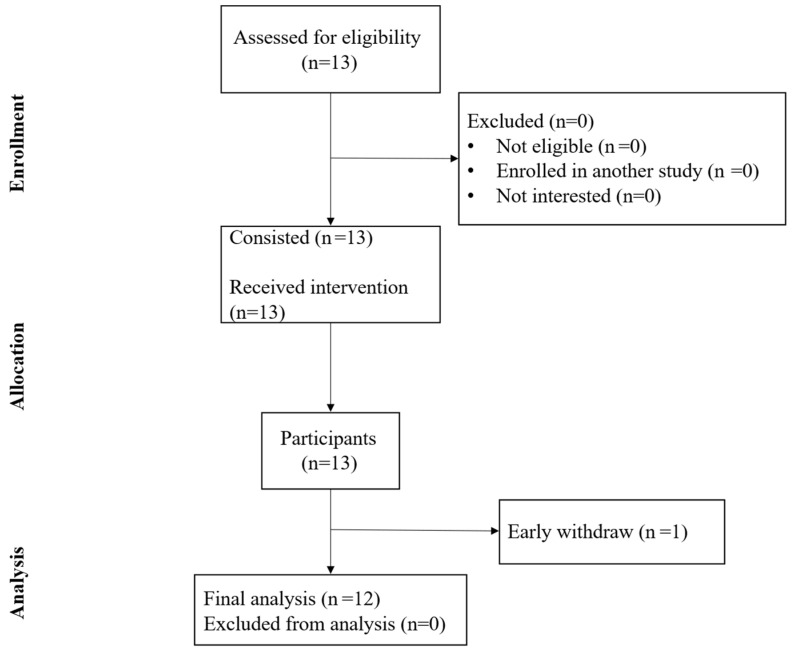
CONSORT diagram.

Twelve (N = 12) healthy individuals (age 21.8 ± 2.2 years, 6 F/6 M) who met the inclusion criteria and agreed to take part in the study were enrolled (Figure 1). The inclusion criteria consisted of adults of both sexes aged over 18 years who were capable of providing informed consent. Exclusion criteria encompassed individuals with a history of psychiatric disorders, sleep disturbances, epilepsy, or any acute or chronic condition that could prevent them from participating in the study.

A CONSORT diagram summarizes recruitment, enrollment, and dropout rates.

### 2.2. Study Design

**Figure 2:**
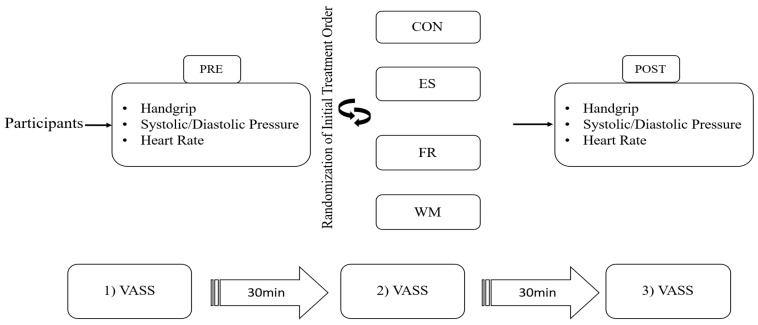
Study flow chart. Subjects assigned in random order in all four different conditions over four continuous weeks. (EEG) = electroencephalography; (CON) = control protocol; (ES) = easy-sleep auto massage auto-program condition; (FR) = fatigue-recovery massage auto-program condition; (WM) = worker-mode massage auto-program condition; VASS: Visual Analog Sleepiness Scale. Start: (1) participants completed the VASS prior to the initiation of the measurement; (2) after massage: participants also completed the VASS exactly 30 min after the transition from the electric automatic massage chair to the bed; (3) after nap: participants also completed the VASS exactly 30 min after waking up from the nap in the bed.

Subjects were randomly assigned into four conditions: (1) Control (CON), in which participants did not receive any massage program, but instead rested for 30 min in an automatic switch-off electric massage chair, followed by another 30 min lying on a standard double bed; (2) easy-sleep (ES), where participants underwent a 30 min preselected massage program and then rested for an additional 30 min on the same double bed; (3) fatigue-recovery (FR), in which participants received a 30 min preselected massage session followed by 30 min lying on the double bed; and (4) worker-mode (WM), where participants completed a 30-min preselected massage program, followed by 30 min of rest on the double bed (Figure 2). All trials were conducted at the same exact time of the day, one week apart, while the order of the various conditions was randomly assigned. To control for factors influencing sleep, participants were instructed to maintain consistent sleep patterns and avoid alcohol, caffeine, and intense physical activity 24 h prior to each session. The data analysts were blinded to the group assignments. All measurements were performed in the same sleep laboratory, at consistent times of day, under controlled conditions with a stable temperature of 22 °C and dim lighting (5 lux). External noise was minimized using white noise at 432 Hz and 55 decibels. The study protocol was approved by the Human Research and Ethics Committee of the University of Thessaly (2334-4/4-2/7 February 2024), and all participants provided written informed consent prior to participation. The trial has been registered as a clinical study at ClinicalsTrials.gov (NCT06784700 – last update 22 January 2025). Available at: https://clinicaltrials.gov/study/NCT06784700. The study was conducted and reported in accordance with the CONSORT guidelines (see Supplementary Materials).

### 2.3. Procedure

All measurements were performed in a sleep lab. Prior to the start of the study, participants completed a Visual Analog Sleepiness Scale (VASS) to evaluate overall sleepiness and health, which was linked to a portable EEG/EOG polysomnography system (HST-mit-tablet, SOMNOmedics, Randersacker, Germany). Each participant then spent 30 min in the automatic massage chair, followed by an additional 30 min lying on a double bed after completing the pre-selected massage session. The sleepiness was assessed at baseline, after the massage session, and after the 30 min “nap time”.

Additionally, at the beginning and end of each condition, participants completed three maximal handgrip attempts to evaluate muscle tone, and their vital signs, including heart rate and blood pressure, were obtained. The study concluded after 60 min of recording. Participants were free to sleep or remain awake during the intervention period.

All experimental sessions were conducted between 9:00 a.m. and 6:00 p.m., with each participant maintaining the same testing time across all four conditions.

### 2.4. Body Composition

Body composition was evaluated using anthropometric measurements, including body mass index (BMI), and assessed through bioelectrical impedance analysis with a Tanita DC-360S device (Tanita Corporation, Tokyo, Japan), following established standard procedures [12].

### 2.5. Visual Scale

The Visual Analog Sleepiness Scale (VASS) was employed to evaluate participants’ levels of sleepiness using a standard 1–10 rating system. Specifically, scores of 0–2 indicated “no sleepiness”, 3–5 corresponded to “moderate sleepiness”, 6–8 reflected “high sleepiness”, and 9–10 represented “very high sleepiness”.

### 2.6. Brain Activity/Sleep Architecture

A portable sleep monitoring system was utilized to evaluate both sleep quality and quantity (Home Sleep Test, SOMNOmedics, GmbH, Germany). The system recorded EEG, EOG, and EMG signals overnight. EEG data were analyzed in 30 s epochs using SOMNOmedics PSG analysis software (Domino panel ver. 3.0.0.8), beginning with automatic analysis, followed by manual editing. PSG was conducted with the HomeSleepTest REM+ device, with electrodes placed according to the 10/20 system at key brain regions: Fp1 (left) and M1 for EEG and near the eyes for EOG. Sleep parameters were reported as follows: Total Sleep Time (total sleep duration during the recording), Sleep Efficiency (Total Sleep Time/Time in Bed), Sustained Sleep Efficiency (Total Sleep Time/[Time in Bed—Stage 2 Sleep Latency]), Sleep Latency (time from lights off to the first epoch of any sleep stage, including N1), N1 Sleep Latency (time from lights off to the start of N1 stage), N2 Sleep Latency (time from lights off to the onset of stage 2), REM Latency (time from sleep onset to first REM stage), Wakes (number of awakenings or transitions to full wakefulness during sleep); and WASO (the total duration (in minutes) of wakefulness occurring after the onset of sleep and before final awakening).

### 2.7. Handgrip Strength Assessment

The handgrip test was employed to evaluate the maximum isometric strength of the dominant hand and arm muscles using a Marsden MG-4800 Hand Dynamometer, and it served as an indicator of muscle tone and alertness [13]. Any decrease in muscle strength following the intervention was interpreted as a reduction in muscle tone.

### 2.8. Massage Session

Automatic Electric Massage Chair (AEMC)

**Figure 3:**
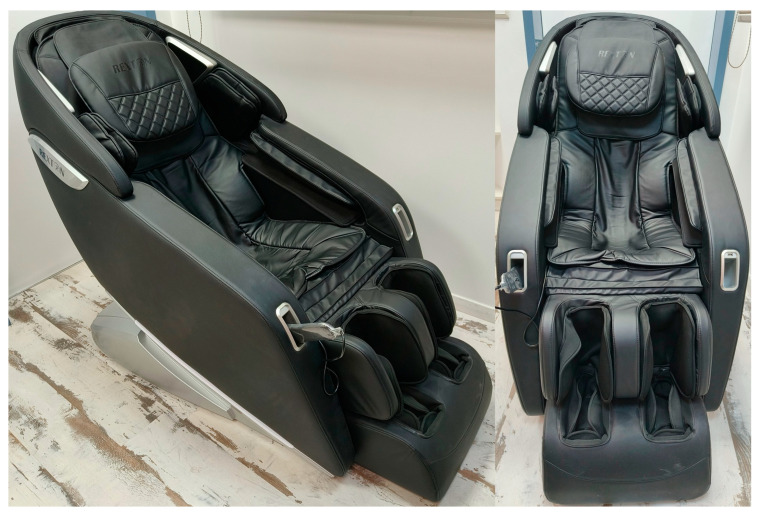
Automatic electric massage chair (model: Z1-SL Full Body 4D).

In this study, a Z1 Full Body 4D Massage Chair (Z1-SL Massage Chair, Volos, Greece, www.back2life.gr) (Figure 3) was utilized. The Z1-SL chair is equipped with a dual S- and L-shaped rail system, enabling massage coverage from the head down to the feet. It incorporates 58 air cushions for compression massage, while 360° rotating mechanical arms adapt to the body’s contour to scan and target treatment zones. Additionally, the device provides twelve pre-set massage programs with varying intensity levels, and its roller system can be adjusted to body widths ranging from 3 to 22 cm to address specific areas, such as the cervical, thoracic, and lumbar spine.

#### 2.8.1. Massage Auto-Programs of the AEMC

The three preselected automatic massage programs were designed by the manufacturer to enhance relaxation, reduce muscle strain, and aid recovery by applying a variety of massage techniques focused on specific muscle areas. The muscle groups that received the massage effect were divided into two main sections (upper and lower body), with each section comprising three subcategories, as described in (Table 1).

#### 2.8.2. Easy-Sleep Program

The easy-sleep (ES) program aims to promote overall body relaxation using slow, gentle motions combined with light air compression, making it suitable for use before bedtime. It incorporates techniques such as kneading, tapping, rolling, and deep tissue massage, targeting specific muscle areas.

#### 2.8.3. Fatigue-Recovery Program

The fatigue-recovery (FR) program is intended to ease muscle tension and reduce fatigue after a demanding day, while improving circulation and aiding in muscle restoration. It employs kneading, tapping, rolling, and deep tissue massage techniques directed at specific muscle areas.

#### 2.8.4. Worker-Mode Program

The worker-mode (WM) program is designed for individuals who spend long periods sitting or performing demanding tasks. It helps relieve stiffness in the shoulders, back, and lower back through kneading, tapping, rolling, and deep tissue massage techniques applied to targeted muscle groups.

### 2.9. Statistical Analysis

The statistical analysis was performed using IBM SPSS Statistics version 29.0 (IBM Corporation, Armonk, NY, USA). An independent samples *t*-test was performed to examine differences in basic characteristics between male and female participants. To evaluate within-subject differences across the four conditions (CON, ES, FR, WM), a General Linear Model (GLM) Repeated Measures ANOVA was applied. Additionally, GLM Repeated Measures ANOVA was used to analyze changes in EEG parameters across the four conditions, followed by Bonferroni post hoc tests to identify pairwise differences. Normality was assessed using the Shapiro–Wilk test and graphical methods, including Normal Q–Q plots, Detrended Q–Q plots, and Box Plots. The significance threshold was set at 0.05. Alongside *p*-values, effect sizes were calculated to determine the magnitude of the observed effects. Given the multiple comparisons performed, the risk of Type I error may increase. Bonferroni post hoc adjustments were applied to reduce this risk, but it should still be considered when interpreting the results.

### 2.10. Power Analysis

Sample size estimations were performed using G*Power 3.1 [14]. A post hoc GLM approach for repeated measures within factors was employed to determine statistical power. The results indicated that with 12 participants, the study achieved a power of 99% for the primary outcome, Sleep Efficiency (effect size = 1.5609, critical F = 2.8915, Ndf = 3, Ddf = 33, power [1-β error prob] = 0.9999).

### 2.11. Trial Registration

The trial was registered under the title “The Usefulness of Automatic Massage Chairs Before Bedtime in Sleep Quality (RoboSleep)” with the full protocol to be accessible through the ClinicalTrials.gov website, trial registration number: NCT06784700 (last updated 22 January 202).

### 2.12. Data Availability

Data for the study are available at ClinicalTrials.gov, with registration number NCT06784700.

## 3. Results

Subject characteristics are presented in Table 2. No statistically significant differences were found between male and female participants, as the groups were equally distributed, and the statistical analysis showed no significant difference (*p* > 0.05).

Pre- and post-intervention assessments among the four different groups are shown in Table 3. Significant differences within groups were observed in handgrip strength (muscle tone) and heart rate, before and after all trials. Additionally, significant differences were found in diastolic pressure during the easy-sleep (ES) and worker-mode (WM) groups (Table 3).

In particular, handgrip strength was reduced after the CON, ES, FR, and WM trials accordingly (%Δ-change: −12.31% ± 11.5; %Δ-change: −11.85% ± 11.5; %Δ-change: −12.42% ± 11.4; %Δ-change: −15.32% ± 9.9, *p* = 0.005). Statistically significant differences were also found among the Δ-change of the aforementioned values.

Moreover, heart rate was reduced after the ES, FR, and WM trials, accordingly (%Δ-change: −21.59% ± 22.8; %Δ-change: −14.69 ± 15.1; %Δ-change: −15.32% ± 9.9, *p* = 0.007). Heart rate decreased mainly after the WM trial (*p* = 0.040). Also, diastolic pressure was reduced after both the ES and WM trials, accordingly (%Δ-change: −7.81% ± 9.9, *p* ≤ 0.005; −11.45 ± 14.0, *p* ≤ 0.005).

A comparative analysis of the VASS across all conditions is presented in Table 4. Sleepiness scores increased significantly from the start to the middle and finish phases across all conditions (*p* ≤ 0.005). Pairwise comparisons using the Bonferroni post hoc test revealed significant differences between VASS scores after the massage programs and after the nap for all conditions (*p* ≤ 0.005).

The sleep parameters are reported in Table 5. A statistically significant effect was observed for sleep efficiency across the four conditions [F(3,33) = 8.978, *p* ≤ 0.001], with the ES condition showing the greatest impact compared to that of the others (*p* ≤ 0.005). The difference (Δ-change) in sleep efficiency between the CON and ES conditions was 5.69% ± 28.3, indicating a notable improvement. Importantly, all participants successfully achieved sleep under each condition. Total Sleep Time (TST) and Sleep Efficiency (SE) were recorded and analyzed (Table 5).

In the ES-massage group, participants reached an average TST of 26.76 ± 11.8 min and SE of 43.44 ± 18.7%. These values confirm that participants entered measurable sleep stages during the nap trials, validating the sleep data collected.

## 4. Discussion

To the best of our knowledge, this is the first study to investigate the effects of automatic electric massage chair protocols on body relaxation and sleep efficiency using objective and subjective measurements.

The main outcome of the current study, when objective measurements of relaxation were used, was that the easy-sleep massage program exerted the most significant effect, reducing heart rate by −21.59% (Table 3). In a subjective scale utilizing the VASS, a significant increase in sleepiness was observed, with the ES massage program showing the most pronounced effect, resulting in a 4.50% increase (Table 4). Furthermore, objective measurements using a PSG–EEG indicated a significant increase in sleep efficiency after the implementation of the ES program compared to the results for the CON, with an improvement of 17.7 min (Table 5). Beyond the expected outcomes of relaxation and improved sleep quality, the ES massage also exerts significant physiological effects on the body, explaining the results observed in the present study. Massage techniques such as kneading, tapping, and deep tissue pressure appear to activate skin and muscle receptors, leading to the stimulation of the parasympathetic nervous system [15]. This activation results in a reduction in heart rate, as evidenced by the observed decrease of −21.59%, and an increase in blood circulation, thereby enhancing the uptake of oxygen and nutrients in the muscles and throughout the body [16]. In addition, massage techniques focusing on the neck and shoulder areas, as indicated in Table 1, may reduce muscle tension in these regions, which are often associated with stress and pain that negatively impact sleep quality [17].

The current study shows that a 30 min full-body ES massage chair program benefits both objective measures and subjective experiences in healthy individuals. The ES protocol incorporates techniques such as kneading, tapping, deep tissue pressure, and rolling, specifically designed to promote muscle relaxation, enhance blood circulation, and alleviate muscular tension. Particularly, the majority of the massage duration, i.e., 22 out of 30 min (73% of the total time), was focused on the cervical (neck) and shoulder regions of the upper-body (Table 1), targeting these areas to maximize the sense of relaxation, comfort, and relief. While there are no directly comparable studies, a study by Hongmin Chu et al., using a subjective scale, assessed the effectiveness of an electric massage chair among office workers, demonstrating that the massage chair effectively alleviated neck and shoulder pain, and a modest improvement was also observed in quality of life scores [18].

Additionally, a significant portion of the total duration of the ES massage program was focused on the area of the heel and foot (70%), (Table 1). A study by Gökbulut et al. found that foot massage applied in menopausal women increases the average daily sleep duration and reduces levels of fatigue and anxiety [19]. Regarding Achilles tendon massage, the most effective approach seems to be the effleurage movements, involving applying stretching motions along the periphery of the Achilles tendon area. According to recent findings, this approach not only effectively reduces pain symptoms in patients with Achilles tendinopathy but also promotes overall muscle relaxation [20,21].

Furthermore, the ES program recorded the largest decrease in heart rate, i.e., −21.55%, indicating a significant parasympathetic activation, promoting relaxation and reducing stress levels, findings that are in agreement with a study by Fu et al. demonstrating a significant reduction in heart rate and improvement in heart rate variability [22]. Regarding the reduction in muscle tone, the WM program demonstrated a substantial reduction in handgrip strength scores by 15%. This is mainly due to the fact that the WM program primarily focuses on the areas of the hands and back, with 21 out of 30 min of massage time allocated to those muscles. Studies on the use of AEMCs are very limited, with only one similar to ours utilizing objective EEG measurements for the assessment of brain activity. In particular, Jeong-Hwan et al. found that 20 min sessions with an AEMC in addition to a binaural sound significantly reduced levels of mental fatigue and improved cognitive function [23].

In another study by Khairudin et al., the use of an AEMC for treating labor pain in primiparous women showed a significant reduction in pain levels, indicating that mechanical massage may serve as a viable, non-invasive alternative approach for pain management during pregnancy and labor [24].

Additionally, massage therapy using an AEMC appears to have a positive effect on chemotherapy-related side effects [25], as well as exhibit some preventive value, particularly in managing musculoskeletal discomfort in populations heavily impacted by work-related musculoskeletal overuse issues [26]. This underscores the importance of incorporating massage and similar interventions as part of a preventive approach for high-risk groups like musicians, alleviating muscle tension and enhancing physical resilience over time.

Another important type of user that benefited from AEMCs is professional athletes. A study by Juncheng Xie et al. [27] demonstrated that the massage performed by an AEMC significantly impacted the reduction of vagus nerve tone and accelerated the elimination of metabolites such as lactate, thereby enhancing recovery from exercise training. These findings suggest that the AEMC may serve as a more efficient tool for rapid athlete recovery, providing stronger recuperative benefits compared to those of other traditional methods.

From a hormonal point of view, there is evidence that massage promotes the release of serotonin [28], while a study by Ji Yeon Baek et al. reported that long-term use of the AEMC can reduce anxiety in adults, as the intervention resulted in significant decreases in cortisol and serum DHEA-S (dehydroepiandrosterone sulfate) levels, along with improvements in mood and health status [29].

It is evident, therefore, that the use of massage chairs can benefit not only people with increased stress or various health issues but also totally healthy individuals in sustaining a healthy lifestyle or promoting a power nap for boosting their cognitive function or improving creativity.

In this study, we recognize several notable strengths, as well as certain unforeseen limitations. The main strengths include the fact that a combination of both objective and subjective methods were employed to evaluate relaxation and brain activity. Additionally, the data analysis was conducted under blinded conditions, with all researchers unaware of the specific session type. Finally, the utilization of a real-time EEG monitoring system offered objective assessment of brain activity, providing valuable insight into the immediate effects of massage on neural function.

One limitation is the small sample size, even though the study had more than 95% power to detect statistically significant differences for the primary outcome. Another important limitation is that we cannot precisely identify the specific muscle groups targeted during the automatic massage session, since there was no use of EMG recording. Furthermore, the menstrual cycle of female participants was not controlled in the study; however, none of the female participants were in the menstrual phase at the time. Additionally, other factors that might influence outcomes, such as changes in participants’ sleep habits or psychological state, were not assessed, and this should be considered as a potential limitation. Finally, while researcher blinding was strictly maintained, we did not assess whether participants could guess the type of massage program utilized, which should be considered when interpreting the results.

## 5. Conclusions

Massage routines such the easy-sleep method, focusing on massaging the cervical and shoulder regions to maximize the sense of relaxation and comfort, are among the most effective protocols for promoting sleep efficiency. These findings support the use of automatic electric massage chairs as a non-pharmacological tool for promoting relaxation and enhancing the feeling of drowsiness. Further research is needed to explore its broader applications in populations with sleep disorders.

## Figures and Tables

**Table 1:** Specification of the four different conditions.

Massage Muscle Groups	CON (min)	(ES) (min)	(FR) (min)	(WM) (min)
**I. Cervical Spine and Shoulders**	-	22	13	14
**II. Back and Hands**	-	18	13	21
**III. Spinal Area**	-	8	11	10
**IV. Gluteal Muscles and** **Biceps Femoris**	-	6	7	11
**V. Gastrocnemius (calf)**	-	17	16	18
**VI. Heels and Feet**	-	20	18	17

Note: CON: control; ES: easy-sleep massage program; FR: fatigue-recovery massage program; WM: worker-mode massage program.

**Table 2:** Baseline characteristics.

Parameters	Values
N	12
Sex	6 M/6 F
Age (years)	21.8 ± 2.2
Body Height (cm)	171.1 ± 9.7
Body Weight (kg)	68.1 ± 9.3
BMI (kg/m^2^)	23.17 ± 1.2
Fat (%)	19.5 ± 6.6
Muscle Mass (kg)	53.2 ± 13.5

Note: Data are presented as Mean ± SD. Significance level: *p* < 0.05; ΒΜΙ: body mass index.

**Table 3:** Pre- and post-intervention assessment among the four different protocols using an electric automatic massage chair.

		CON			ES			FR			WM	
Variables	Pre (95% CI)	Post (95% CI)	*p* Value (Cohen’s d)	Pre (95% CI)	Post (95% CI)	*p* Value (Cohen’s d)	Pre (95% CI)	Post (95% CI)	*p* Value (Cohen’s d)	Pre (95% CI)	Post (95% CI)	*p* Value (Cohen’s d)
			(Δ-Change%)			(Δ-Change%)			(Δ-Change%)			(Δ-Change%)
**N**	12	12	-	12	12	-	12	12	-	12	12	-
**Systolic Pressure** **(mmHg)**	128.6 ± 22.4 (116 to 141)	119.4 ± 10.4 (114 to 125)	0.118 (0.526) (−7.19% ± 24.7)	116.9 ± 9.3 (112 to 122)	116.3 ± 11.5 (110 to 123)	0.840 (0.057) (−0.50% ± 14.8)	123.4 ± 13.6 (116 to 131)	118.0 ± 13.7 (110 to 126)	0.076 (0.395) (−4.32% ± 19.3)	123.0 ± 14.0 (115 to 131)	118.1 ± 12.0 (111 to 125)	0.116 (0.375) (−3.99% ± 18.4)
**Diastolic Pressure** **(mmHg)**	72.7 ± 9.9 (67.1 to 78.3)	66.9 ± 8.5 (62.1 to 71.7)	0.076 (0.628) (−8% ± 13.0)	70.3 ± 7.2 (65.7 to 74.9)	64.8 ± 6.7 (61 to 68.6)	0.004 (0.790) (−7.81% ± 9.9)	71.8 ± 12.7 (64.6 to 79)	64.9 ± 9.3 (59.6 to 70.2)	0.051 (0.619) (−9.62% ± 15.8)	71.3 ± 10.2 (65.5 to 77.1)	63.1 ± 9.6 (57.7 to 68.5)	0.003 (0.827) (−11.45% ± 14.0)
**Heart Rate** **(beats/min)**	74.0 ± 10.0 (68.3 to 79.7)	75.4 ± 16.9 (65.8 to 85)	0.729 (0.100) (1.81% ± 19.7)	85.7 ± 22.1 (73.2 to 98.2)	67.2 ± 5.8 (63.9 to 70.5)	0.007 (1.145) (−21.59% ± 22.8)	75.3 ± 12.6 (68.1 to 82.5)	64.2 ± 8.3 (59.5 to 68.9)	0.000 (1.040) (−14.69% ± 15.1)	74.1 ± 9.3 (68.8 to 79.4)	63.4 ± 8.7 (58.5 to 68.3)	0.001 *** (1.118) (−14.49 ± 12.8)
**Handgrip Strength** **(kg)**	34.3 ± 8.8 (28.4 to 38.4)	30.1 ± 7.4 (25.9 to 34.3)	0.001 (0.516) (−12.31% ± 11.5)	34.2 ± 8.7 (21.7 to 46.7)	30.1 ± 7.5 (25.9 to 34.3)	0.008 * (0.504) (−11.85% ± 11.5)	33.4 ± 7.2 (29.3 to 37.5)	29.3 ± 8.7 (24.4 to 34.2)	0.019 (0.513) (−12.42% ± 11.4)	33.4 ± 7.5 (29.2 to 37.6)	28.3 ± 7.1 (24.3 to 32.3)	0.002 ** (0.698) (−15.32% ± 9.9)

Note: Data are presented as Mean ± SD. Significance level: *p* < 0.05; CON: control; ES: easy-sleep massage program; FR: fatigue-recovery massage program; WM: worker-mode massage program; Δ-Change: % difference compared to pre values; Paired *t*-Test: means of pre–post measurements between (CON vs. ES vs. FR vs. WM) protocols. * Differences between the Δ-changes were assessed using a GLM Repeated Measures test; Heart Rate: CON vs. ES vs. FR vs. WM, *p* = 0.007. ** Differences between the Δ-changes were assessed using an ANOVA test; CON vs. WM, *p* = 0.040 (Bonferroni post hoc test). *** Differences between protocols (CON vs. ACT vs. REL) were assessed using a GLM Repeated Measures test; Heart Rate: [F(3,33) = 5.503, *p* = 0.015], specifically for post-CON vs. post-WM.

**Table 4:** VASS across three phases for the four automatic electric massage chair protocols.

Sleepiness Scale	Baseline	After Massage Chair (Δ-Change%)	After Nap (Δ-Change%)	*p* Value
**N**	12	12	12	-
**(CON)**	2.91 ± 2.0	5.66 ± 2.5 *	7.00 ± 2.0 *	<0.001
		(2.75% ± 0.5)	(4.09% ± 0.0)	
**(ES)**	3.08 ± 1.8	5.83 ± 1.8 *	7.58 ± 2.3 *	<0.001
		(2.75% ± 0.0)	(4.50% ± 0.5)	
**(FR)**	3.25 ± 1.7	5.91 ± 2.1 *	7.66 ± 2.2 *	<0.001
		(2.66% ± 0.4)	(4.41% ± 0.5)	
**(WM)**	4.08 ± 2.1	6.75 ± 1.5 *	7.75 ± 2.2 *	<0.001
		(2.67% ± 0.6)	(3.67% ± 0.1)	

Note: Data are presented as Mean ± SD. Significance level: *p* < 0.05; CON: control; ES: easy-sleep massage program; FR: fatigue-recovery massage program; WM: worker-mode massage program. * Differences between pairwise comparisons were assessed using an ANOVA test. CON: start phase vs. middle phase, *p* < 0.001, and start phase vs. finish phase, *p* = 0.000 (Bonferroni post hoc test); EA: start phase vs. middle phase, *p* < 0.001, start phase vs. finish phase, *p* = 0.000, and middle phase vs. finish phase, *p* = 0.004 (Bonferroni post hoc test); FR: start phase vs. middle phase, *p* < 0.001, start phase vs. finish phase, *p* < 0.001, and middle phase vs. finish phase, *p* = 0.017 (Bonferroni post hoc test); WM: start phase vs. middle phase, *p* = 0.000, and start phase vs. finish phase, *p* = 0.002 (Bonferroni post hoc test). The Δ-changes were assessed using a GLM Repeated Measures test, revealing significant differences in sleepiness scores across phases.

**Table 5:** Sleep parameters among the four different conditions.

Variables	CON (95% CI)	ES (95% CI)	FR (95% CI)	WM (95% CI)	*p* Value (Effect Size: Partial η^2^)
**N**	12	12	12	12	-
**TST** **(min)**	20.60 ± 12.5	26.76 ± 11.8 *	25.74 ± 13.8	25.06 ± 13.6	0.239
	(13.5 to 27.7)	(20.1 to 33.4)	(17.9 to 33.5)	(17.4 to 32.8)	(0.121)
**Sleep Efficiency** **(%)**	37.75 ± 21.2	43.44 ± 18.7 *	25.74 ± 13.8	25.06 ± 13.6	<0.001
	(25.8 to 49.8)	(32.8 to 54)	(17.9 to 33.5)	(17.4 to 32.8)	(0.449)
**Sustained Sleep Efficiency** **(%)**	41.88 ± 18.5	49.74 ± 17.9	39.45 ± 25.0	42.03 ± 21.0	0.413
	(31.4 to 52.4)	(39.6 to 59.8)	(25.4 to 53.6)	(30.1 to 53.9)	(0.079)
**Sleep Latency** **(min)**	8.43 ± 13.2	8.36 ± 10.3	3.79 ± 5.0	7.47 ± 12.7	0.556
	(0.96 to 15.9)	(2.53 to 14.2)	(0.96 to 6.62)	(0.28 to 14.7)	(0.049)
**Sleep Latency N1** **(min)**	8.75 ± 13.1	9.53 ± 10.3	7.28 ± 9.0	8.10 ± 12.9	0.864
	(1.34 to 16.2)	(3.7 to 15.4)	(2.19 to 12.4)	(0.8 to 15.4)	(0.010)
**Sleep Latency N2** **(min)**	17.73 ± 15.4	11.25 ± 11.3	7.93 ± 13.4	16.31 ± 14.5	0.306
	(9.02 to 26.4)	(4.86 to 17.6)	(0.35 to 15.5)	(8.11 to 24.5)	(0.123)
**Wakes** **(index)**	8.91 ± 3.36	9.75 ± 3.4	8.91 ± 4.1	8.75 ± 4.4	0.861
	(7.01 to 10.8)	(7.83 to 11.7)	(6.59 to 11.2)	(6.26 to 11.2)	(0.018)
**Wake > 3 min** **(index)**	2.09 ± 0.8	2.09 ± 1.1	2.45 ± 1.6	2.72 ± 1.5	0.587
	(1.64 to 2.54)	(1.47 to 2.71)	(1.55 to 3.36)	(1.87 to 3.57)	(0.054)
**WASO** **(Wake After Sleep Onset)** **(min·s)**	30.25 ± 11.1	26.79 ± 8.1	27.04 ± 10.5	29.4 ± 12.7	0.803
	(24 to 36.5)	(22.2 to 31.4)	(21.1 to 33)	(22.2 to 36.6)	(0.032)

Note: Data are presented as Mean ± SD. Significance level: *p* < 0.05; CON: control; ES: easy-sleep massage program; FR: fatigue-recovery massage program; WM: worker-mode massage program; TST: total sleep time. * Differences between pairwise comparisons were assessed using an ANOVA test; TST: CON vs. ES, *p* = 0.050; Sleep Efficiency: ES vs. FR, *p* = 0.014 and ES vs. WM, *p* = 0.003 (Bonferroni post hoc test). Total duration of recording: 60 min.

## Data Availability

The original contributions presented in the study are included in the article; further inquiries can be directed to the corresponding author.

## Supplementary Materials

The following supporting information can be downloaded at: https://www.mdpi.com/article/10.3390/healthcare13182291/s1, CONSORT checklist.
